# Evaluation of a Mobile Farmer's Market Aimed at Increasing Fruit and Vegetable Consumption in Food Deserts: A Pilot Study to Determine Evaluation Feasibility

**DOI:** 10.1089/heq.2018.0003

**Published:** 2018-12-18

**Authors:** Tiffany L. Gary-Webb, Todd M. Bear, Dara D. Mendez, Mary D. Schiff, Ehrrin Keenan, Anthony Fabio

**Affiliations:** ^1^ Department of Behavioral and Community Health Sciences, University of Pittsburgh Graduate School of Public Health, Pittsburgh, Pennsylvania.; ^2^ Department of Epidemiology, University of Pittsburgh Graduate School of Public Health, Pittsburgh, Pennsylvania.; ^3^ Greater Pittsburgh Community Food Bank, Pittsburgh, Pennsylvania.

**Keywords:** evaluation, food deserts, mobile market, poverty

## Abstract

**Purpose:** In November 2015, Greater Pittsburgh Community Food Bank implemented a pilot phase of the *Green Grocer* mobile market, a program aimed at improving access to locally sourced fresh foods in low-resource neighborhoods. We conducted an evaluation of this pilot phase.

**Methods:** We conducted baseline surveys of residents in six neighborhoods that received *Green Grocer* in the pilot phase to understand the food environment, including perceptions of fresh food availability, and another survey of *Green Grocer* consumers to evaluate their experiences and satisfaction. We measured respondent intake of fruit and vegetable in the terms of days per week and servings per day. We used Poisson regression with cluster-robust standard errors to model the average change in produce consumption pre–post intervention.

**Results:** Residents of select communities observed meaningful improvements in intake. After covariate adjustment, Homewood residents observed an average 13% increase in vegetable intake (days/week) rates post-*Green Grocer* (*p*=0.04). Clairton residents also showed marked increases, with an average 20% increase in vegetable intake (servings/day) (*p*=0.049). After 6 months, declines in produce purchase from discount stores and supercenters were observed alongside increases in procurement from *Green Grocer*, farmer's markets, gardens, and other sources.

**Conclusion:** Our preliminary work provides support that this mobile market serving under-resourced areas was valued by consumers and showed increases in vegetable consumption in several neighborhoods. When scaled-up, this program had the potential to reduce geographically-based food and health disparities.

## Introduction

There are significant disparities in the prevalence of overweight and obesity in the United States among communities of color and low-income communities compared with their White counterparts. For example, the rates of obesity in 2011–2014 were 48.1% among Non-Hispanic Blacks and 42.5% among Hispanics compared with 34.5% among non-Hispanic Whites.^1–3^ Similarly, disparities in rates of obesity are seen among those with lower incomes and less education, particularly among women.^4^ Many potential solutions to curb the obesity epidemic have been promoted, but most have focused on individual-level behavior change strategies, which have had limited impact on improving population-based rates. Even after adjusting for individual-level socioeconomic status, residence in a disadvantaged neighborhood is associated with increased rates of obesity,^5^ and data suggest that these neighborhoods have fewer resources available to promote healthy behaviors and facilitate good health. Obesity (and other poor health outcomes such as diabetes and cardiovascular disease) in disadvantaged neighborhoods may be manifested through aspects of the physical environment such as lack of stores providing healthy foods.^6–13^ Thus, the logical next steps to address obesity are to conduct community-level, systemic interventions aimed at modifying “unhealthy” environments by increasing access to needed resources that promote healthy behaviors in the communities and among the individuals most in need.

The recent emphasis on how aspects of the social and built environment might contribute to racial and socioeconomic disparities in dietary behaviors and obesity shows promise in understanding the potential structural or neighborhood-level influences on the epidemic.^7,13^ For instance, there is evidence from a number of studies that communities of color and marginalized populations live in neighborhood environments with limited food stores, hazards, and aesthetic problems such as vacant houses, traffic, and crime.^7^ These neighborhood environments have been shown to be independently associated with individuals' diet quality.^13^ Specifically, studies found positive associations between access to healthy foods (more supermarkets and fewer convenience stores) in a neighborhood and better health behaviors/lower rates of obesity.^13^ A consistent trend is that low-income, minority residents have poor access to supermarkets and healthy foods while also facing abundant access to fast food outlets and energy-dense foods.^7,8,13^ No doubt, this explosion of research over the last several decades on the built environment^5^ and food environments,^13^ in particular, has enhanced our understanding of the complex causes of the obesity epidemic.

The concept of the “food desert,” defined as neighborhoods that do not have ready access to fresh, healthy, affordable food, has now become a term/concept that is understood and endorsed by individuals and community groups. However, new studies are needed that contribute to the knowledge base to inform successful individual and community-based changes. Further, new studies are showing that just the introduction of a grocery store into these communities is not enough. For example, one study that introduced a supermarket into a low-resource neighborhood found improvements in dietary quality and perceived access to healthy food, but no difference in fruit and vegetable consumption compared with a similar control neighborhood.^14^ Research on farmer's markets suggests that low-income women were increasingly willing to shop at farmer's markets when the price savings increased and the market was incrementally closer to their residence.^15^ Mobile food markets are a promising strategy to combat the negative effects of food deserts because they expand access to a larger area, yet few formal evaluation studies have been conducted to understand whether this approach is effective.

Mobile food markets are a relatively recent phenomenon, and studies on them are scarce and have produced mixed results.^16–18^ Several studies have shown an increase in fruit and vegetable consumption among targeted populations, including children and seniors,^19,20^ while others have shown modest uptake and effects.^16^ Despite studies showing positive outcomes related to accessible fresh produce, obstacles remain (e.g., affordability, reaching intended customers, timing, lack of variety, and lack of advertising). A focus group study^16^ that interviewed customers of mobile markets in four sites across the United States outlined recommendations to increase mobile market use in low access areas, including emphasizing convenience of locations and payment options, providing a variety of food options, and providing cooking demonstrations and events.

Greater Pittsburgh Community Food Bank (GPCFB) piloted the *Green Grocer* mobile farmer's market starting in November 2015 in collaboration with our team. This “farm stand on wheels” is a delivery truck of refrigerated, ready-to-sell fresh fruits and vegetables, and other healthy food options that visited six locations in Allegheny County, PA (which includes the City of Pittsburgh), and is one of a few mobile markets in the country owned and operated by a food bank. *Green Grocer* stops are scheduled in one to three neighborhoods per day for a total of 1 h each. The truck is staffed by a full-time manager/driver who is assisted by ride-along volunteers (some of whom are in the AmeriCorps™ program). The program is designed to be affordable and accepts multiple forms of payment, including Supplemental Nutrition Assistance Program (SNAP)/EBT. GPCFB researched prices through the U.S. Department of Agriculture (USDA) fair market value, local grocery stores, etc., and made *Green Grocer* pricing very competitive. They are not always able to beat supermarket prices (because of economy of scale), but they often do. Further, since the market stops are in areas where grocery stores are less accessible, they are not competing with local retailers. Their standard markup is 30%, but they adjust that based on research, the perishability of the product, etc. In addition, the market sells frozen meats and dry goods (e.g., white and brown rice, various pastas, dry beans, oats), so that customers can create complete meals. *Green Grocer* distributes recipe cards and other nutrition education information to assist customers in building healthy, nutritious meals. Other items being considered are cheese, eggs, and other supplemental items such as honey, nuts, canned goods, and seed packets. The goals of the program are as follows: (1) increase access to high-quality, healthy, and affordable food in low-resource communities; (2) increase long-term sustainability of the program; and (3) provide a high-quality consumer experience to ensure long-term patronage.

We identified target areas for the *Green Grocer* mobile market using five key constructs: poverty/income rates, SNAP usage, obesity rates, grocery/supermarket access, and mass transit access. We used data from the U.S. Census/American Community Survey (ACS), Allegheny County Health Department, the City of Pittsburgh, USDA, and the Port Authority of Allegheny County General Transit Feed. The thresholds for poverty and SNAP usage were based on national averages using the 2009–2013 ACS, 15.8% and 12.4%, respectively. High obesity rates were defined as >25% prevalence within the census tract. Low grocery access was defined as areas over ½ mile from a grocery store. Low transit access was defined as areas served by less than four bus trips per hour during the weekday within ¼ mile, a 5-min walk. Census tracts were excluded with small population sizes (i.e., <100 total population). The GPCFB asked that we pay special attention to areas where there were senior high rises, senior centers, and women infant and children program (WIC) offices. Senior high rises and centers were targeted because of the high rates of food insecurity among the elderly. WIC offices were of importance because this is where the Farmer's Market Nutrition Program coupons are distributed. Most of the data on locations of WIC offices, senior high rises, and senior centers were in PDF formats that had to be manually added to a database and geocoded. We performed an overlay analysis to determine geographic locations of greatest need by looking at the intersection of tracts that had high rates of poverty, high SNAP usage, and high rates of obesity first. We then narrowed down the target areas by taking into account the areas with low grocery store and transit access.

In this article, we briefly describe the pilot-phase evaluation of the *Green Grocer* mobile market. After the GPCFB implemented the mobile food market in the first neighborhood, we collaborated with them and completed a preliminary pilot neighborhood-level survey in that community in December 2015. As the *Green Grocer* pilot program was implemented in new neighborhoods, we completed additional baseline surveys (*n*=6 neighborhoods total). We also developed and completed surveys of the *Green Grocer* consumers starting in January 2016. Overall, the pilot phase was implemented by GPCFB from November 2015 to July 2016. We conducted 6 months follow-up surveys from August 2016 to January 2017, and this article describes the outcome of this evaluation. As this was an evaluation of a real-world program being implemented in priority areas, we did not have the opportunity to randomize neighborhoods to receive the *Green Grocer* mobile market or not. Thus, we elected for a pre–post design to capture changes over time.

## Methods

### Baseline pilot surveys for target neighborhoods and consumers of the *Green Grocer* mobile market program

To test the feasibility and success of the *Green Grocer* mobile market, the Office of Health Survey Research at the University of Pittsburgh conducted two pilot surveys for evaluation. The community-wide survey (CWS) recruited households (*n*=267 pre and *n*=223 post) in six neighborhoods. The goal of this initial data collection was to obtain baseline measures for the intervention neighborhoods. The Customer Satisfaction and Outcomes Survey (CSOS) recruited *Green Grocer* customers (*n*=102 pre and 48 post) who visited the mobile markets one or more times during the survey period. The goal of CSOS was to assess individual-level dietary patterns, behaviors, and perception of food access.

For the CWS, we used address-based sampling (ABS) to select Census block groups that were within a 1-mile radius of the truck's visit location. From each of these service areas, we sampled ∼500 addresses. We mailed postcards to the sampled addresses describing the survey and inviting an adult member of each household to participate in the survey. We used a mixed-mode approach (i.e., web and phone) to decrease participant burden and increase response rate. Interested residents could choose to complete the survey through the web using Qualtrics (a web-based survey system) or by calling our survey center to complete the survey using our Computer-Assisted Telephone Interviewing System. When telephone numbers were available from the ABS sample, interviewers made call attempts to potential respondents after 1-week post mailing. As an incentive and to compensate participants for their time, participants who completed the CWS were entered into a lottery with a chance of winning US$150 in gift cards. CWS follow-up began in November 2016 and ceased in January 2017.

For the CSOS, a convenience sample of *Green Grocer* customers was recruited by the GPCFB staff, and contacted and followed over 6 months to obtain measures of satisfaction with the *Green Grocer* market and to track changes in diet-related behavior and perception of food access over time. Participants again had the option to complete the survey through web or over the phone, and each participant received a US$5 voucher to use at the *Green Grocer* mobile market. As this was a real-world evaluation of an existing program and did not fit guidelines for human subjects research, we were not required to obtain IRB approval.

### Analysis strategy

We evaluated pre–post changes in fruit and vegetable consumption, along with neighborhood perceptions of food access, within five targeted neighborhoods for which we had follow-up (one site was discontinued due to low sales and low customer interaction). We based neighborhood inclusion upon a set of criteria that identified poverty/income level, obesity rate, SNAP usage, grocery access, and transit access. We set the alpha level at 0.05 and used SAS v9.4 (Cary, NC) for all analyses.

### Community-wide survey

#### Variable descriptions

Our exposure, the intervention, was a binary variable with indicator levels “pre” and “post.” For this CWS, our primary outcomes of interest were measures of fruit and vegetable consumption. Respondents reported intake in terms of “days per week” and “servings per day” for both fruit and vegetables using the widely administered measure from the Behavioral Risk Factor Surveillance System, a population-based survey conducted by the Centers for Disease Control and Prevention. As such, four consumption metrics were produced from count data responses. Our secondary outcomes of interest were metrics evaluating neighborhood food environment. We gauged respondent perceptions of the availability, quality, and selection of local produce through Likert-scale responses. While we originally had five categories, we later collapsed these responses into dichotomous variables, comparing “strongly agree, agree, or neutral” with “disagree or strongly disagree.” We created an additional binary (y/n) metric using USDA standard intake guidelines (recommending daily intake: 5+ fruits or vegetables).^21^ In alignment with our evaluation aims, our neighborhood variable consisted of the following communities for which we had adequate follow-up: “California-Kirkbride,” “Clairton,” “Homewood,” “Mt. Oliver,” and “Wilmerding.” Neighborhood characteristics are described elsewhere (manuscript under review). We included the following individual-level demographic factors in our analysis: gender, race, age, marital status, annual household income, and education level. We also included a covariate describing the seasonality of responses as the intervention was implemented at varying times for different neighborhoods.

#### Analytic approach

We computed descriptive statistics for our sample, both overall and for each neighborhood. We calculated means and standard deviations for numeric and count variables, and frequency and sample categorical measures. We conducted bivariate analyses, by neighborhood, to determine if the intervention was associated with pre–post changes in select outcome measures (such as fruit and vegetable consumption and neighborhood perception metrics). We determined the significance of pre–post differences in the means of numeric variables and percentages of categorical variables using the Kruskal–Wallis and chi-squared tests, respectively.

We modeled each consumption metric separately, yielding a total of four unique models. Given the count nature of our outcomes, we used Poisson regression with a log link to model the average change in produce consumption pre–post intervention. While equality of mean and variance is assumed under the Poisson distribution, the variance of our select outcome measures was often much smaller than the mean. To account for violation of this assumption, and prevent bias of standard errors due to dispersion, we obtained cluster-robust standard errors using generalized estimating equations.^22,23^ To obtain neighborhood-specific results, we introduced an interaction between our binary exposure and neighborhood variable. We utilized a backward step-wise selection approach, in which covariates were eliminated on a singular basis, which were not significantly contributing to the model at the prespecified 0.20 level.^24,25^ Given our *a priori* evaluation purpose, we included our main exposure, neighborhood variable, as well as their interaction in all models, regardless of significance. Both unadjusted and multivariable models were fitted for each consumption metric, with crude and final model results displayed in Table 3. We produced rate ratios and their 95% confidence intervals for each outcome measure, and assessed their individual significance using the Wald chi-squared tests. We conducted sensitivity analyses by modeling each unique neighborhood–outcome pair individually for a total of 20 separate models (5 neighborhoods×4 outcome metrics).

### Consumer Satisfaction and Outcomes Survey

#### Variable descriptions

Again, our exposure was the intervention pre–post. For this consumer-based survey, our outcomes of interest were metrics evaluating satisfaction with the *Green Grocer* mobile market. We gauged respondent perceptions of the affordability, quality, and selection of *Green Grocer* items through Likert-scale responses. While we originally had five categories, we later collapsed these responses into “strongly agree,” “agree,” and “neutral, disagree, or strongly disagree” for each of the three perception metrics. We created a four-level nominal variable based upon typical produce purchasing habits of respondents. Additional details can be found in Table 4.

#### Analytic approach

As the CSOS was administered at the individual level, we restricted our analysis to those with both pre- and post data (*n*=48). We conducted bivariate analyses to test for the significance of pre–post changes in mobile market satisfaction metrics. To account for use of paired responses, we determined the significance of all pre–post changes using Bowker's test of symmetry. Bowker's test is an extension of the McNemar test that can accommodate variables with more than two response categories.^26^

## Results

### Community-wide survey

#### Descriptive statistics

Overall response rates for the CWS were 28% and 22% for pre- and postsurveys, respectively, as defined by the American Association for Public Opinion Research (AAPOR).^27^ Our sample was predominantly female (74%), non-Hispanic White (56%), with a mean age of 61 years. However, neighborhood composition was not uniform, as demographic characteristics varied by community. Given our *a priori* evaluation aims, we produced neighborhood-specific descriptive statistics, which are included in Table 1. With the exception of Homewood (*n*=111), neighborhood samples were nearly identical in size (*n*=95). The majority of Mt. Oliver (72%) and Wilmerding (95%) residents were non-Hispanic White, while 72% of Homewood respondents were non-Hispanic Black. Resident marital status and educational attainment also varied by neighborhood. Many respondents from California-Kirkbride (37%) and Mt. Oliver (35%) were unmarried, whereas most residents from Clairton (40%), Homewood (36%), and Wilmerding (43%) were married. A greater proportion of respondents from California-Kirkbride (41%) and Homewood (34%) were college graduates, while the majority of Wilmerding residents (46%) were high school graduates or General Educational Development recipients. Respondent age was relatively uniform across neighborhoods, with averages ranging from 57 years in Mt. Oliver to 63 years in Clairton.

**Table 1.** Distribution of Sample Characteristics Overall and by Neighborhood (Community-Wide Survey, 2015–2017)

Sample characteristics	*n* (%) or mean (SD)					
	Overall (*n*=490)	California-Kirkbride (*n*=94)	Clairton (*n*=95)	Homewood (*n*=111)	Mt. Oliver (*n*=95)	Wilmerding (*n*=95)
Female, *n* (%)	364 (74.3)	70 (74.5)	76 (80.0)	83 (74.8)	73 (76.8)	62 (65.3)
Race, *n* (%)						
Non-Hispanic White	271 (56.2)	42 (46.7)	46 (48.9)	26 (23.9)	68 (71.6)	89 (94.7)
Non-Hispanic Black	190 (39.4)	42 (46.7)	45 (47.9)	78 (71.6)	21 (22.1)	4 (4.3)
Hispanic and other	21 (4.4)	6 (6.7)	3 (3.2)	5 (4.6)	6 (6.3)	1 (1.1)
Annual household income, *n* (%)						
<$15,000	95 (22.0)	19 (23.8)	15 (19.0)	24 (24.5)	23 (26.7)	14 (15.7)
$15,000 to <$25,000	126 (29.2)	19 (23.8)	29 (36.7)	28 (28.6)	28 (32.6)	22 (24.7)
$25,000 to <$50,000	117 (27.1)	27 (33.8)	22 (27.9)	13 (13.3)	21 (24.4)	34 (38.2)
≥$50,000	94 (21.8)	15 (18.8)	13 (16.5)	33 (33.7)	14 (16.3)	19 (21.4)
Marital status, *n* (%)						
Married	168 (34.9)	24 (26.4)	38 (40.0)	39 (35.8)	26 (28.3)	41 (43.2)
Divorced/separated	93 (19.3)	20 (22.0)	14 (14.7)	19 (17.4)	19 (20.7)	21 (22.1)
Widowed	94 (19.5)	13 (14.3)	19 (20.0)	29 (26.6)	15 (16.3)	18 (19.0)
Unmarried	127 (26.4)	34 (37.4)	24 (25.3)	22 (20.2)	32 (34.8)	15 (15.8)
Educational attainment, *n* (%)						
Less than high school	34 (7.0)	11 (11.8)	5 (5.3)	10 (9.0)	1 (1.1)	7 (7.4)
High school graduate/GED	180 (36.8)	23 (24.7)	43 (45.3)	33 (29.7)	37 (39.0)	44 (46.3)
Some college	156 (31.9)	21 (22.6)	36 (37.9)	30 (27.0)	42 (44.2)	27 (28.4)
College graduate or more	119 (24.3)	38 (40.9)	11 (11.6)	38 (34.2)	15 (15.8)	17 (17.9)
Season of response, *n* (%)						
November, December	203 (48.2)	33 (39.8)	47 (60.3)	44 (43.1)	34 (46.6)	45 (52.9)
January, March, April	218 (51.8)	50 (60.2)	31 (39.7)	58 (56.9)	39 (53.4)	40 (47.1)
Age (years), mean (SD)	61.0 (14.9)	58.7 (15.9)	63.3 (14.0)	63.2 (13.9)	57.3 (16.1)	62.7 (13.9)

SD, standard deviation; GED, General Educational Development test.

#### Individual fruit and vegetable consumption

Unadjusted estimates of our primary outcomes of interest, both pre- and post-intervention, are displayed in Table 2 for each target neighborhood and for all neighborhoods combined. Crude rates of produce consumption varied by neighborhood, with few pre–post differences proving statistically significant. Generally speaking, California-Kirkbride and Wilmerding had the lowest rates of fruit and vegetable intake at baseline. Most measures of intake remained stable pre–post for California-Kirkbride residents, while modest increases and decreases varied, and were unique to each subsequent neighborhood. Overall, all neighborhoods showed declines in daily servings of fruit. With regard to daily vegetable intake per week, Homewood residents reported moderate increases, those from Wilmerding reported no change in this metric, and Mt. Oliver respondents reported significant decreases. Clairton residents, however, observed modest increases in both fruit (days/week) and vegetable (servings/day) consumption pre–post. In fact, mean servings per day of vegetables was lowest for Clairton residents at baseline (at 1.8 servings), and increased significantly to 2.2 servings post-intervention (*p*=0.02).

**Table 2.** Distribution of Produce Consumption and Perception Metrics by Intervention Time, Overall, and by Neighborhood (Community-Wide Survey, 2015–2017)

Sample characteristics	Mean (SD) or *n* (%)																	
	Overall			California-Kirkbride			Clairton			Homewood			Mt. Oliver			Wilmerding		
	Pre (*n*=267)	Post (*n*=223)	*p*^a^	Pre (*n*=50)	Post (*n*=44)	*p*^a^	Pre (*n*=51)	Post (*n*=44)	*p*^a^	Pre (*n*=58)	Post (*n*=53)	*p*^a^	Pre (*n*=51)	Post (*n*=44)	*p*^a^	Pre (*n*=57)	Post (*n*=38)	*p*^a^
Individual produce consumption																		
Days per week eating fruit, mean (SD)	4.6 (2.3)	4.4 (2.2)	0.34	4.1 (2.5)	4.1 (2.2)	0.92	4.5 (2.3)	4.7 (2.3)	0.49	5.2 (2.2)	4.8 (2.2)	0.31	4.9 (2.1)	4.4 (1.9)	0.22	4.1 (2.3)	3.6 (2.1)	0.35
Servings per day of fruit, mean (SD)	1.8 (1.2)	1.6 (0.9)	0.05	1.8 (1.4)	1.5 (0.8)	0.24	1.9 (1.4)	1.7 (1.0)	0.62	1.9 (1.2)	1.8 (1.0)	0.77	1.8 (1.1)	1.6 (0.9)	0.21	1.6 (0.7)	1.4 (0.7)	0.13
Days per week eating vegetables, mean (SD)	5.5 (1.8)	5.4 (1.8)	0.33	5.3 (2.0)	5.3 (1.9)	0.67	5.4 (1.8)	5.0 (2.0)	0.34	5.7 (1.8)	6.0 (1.5)	0.41	5.8 (1.7)	5.2 (1.7)	0.04	5.3 (1.7)	5.3 (1.6)	0.98
Servings per day of vegetables, mean (SD)	2.0 (1.3)	2.0 (1.0)	0.85	2.0 (1.2)	2.0 (1.2)	0.66	1.8 (1.0)	2.2 (1.0)	0.02	2.2 (1.8)	2.0 (1.1)	0.98	2.0 (1.0)	1.9 (0.9)	0.84	2.0 (1.2)	1.7 (0.7)	0.15
Met USDA^b^ intake guidelines, *n* (%)	37 (15)	26 (12.3)	0.41	8 (16.3)	5 (12.5)	0.61	6 (12.5)	5 (12.2)	0.97	13 (24.5)	9 (17.7)	0.39	6 (12.2)	5 (11.9)	0.96	4 (8.3)	2 (5.4)	0.60
Neighborhood perceptions of food environment																		
Easy access/purchase of produce, *n* (%)																		
Strongly agree, agree, or neutral	195 (73.9)	162 (73)	0.82	39 (78.0)	33 (75.0)	0.73	16 (32.7)	13 (30.2)	0.66	48 (82.8)	45 (84.9)	0.76	43 (86.0)	39 (88.6)	0.70	49 (86.0)	32 (84.2)	0.81
Disagree or strongly disagree	69 (26.1)	60 (27)		11 (22.0)	11 (25.0)		33 (67.4)	30 (69.8)		10 (17.2)	8 (15.1)		7 (14.0)	5 (11.4)		8 (14.0)	6 (15.8)	
High-quality produce, *n* (%)																		
Strongly agree, agree, or neutral	196 (75.4)	161 (73.2)	0.58	39 (79.6)	30 (71.4)	0.37	19 (39.6)	19 (44.8)	0.66	50 (87.7)	44 (83.0)	0.49	40 (81.6)	34 (77.3)	0.60	48 (84.2)	34 (89.5)	0.47
Disagree or strongly disagree	64 (24.6)	59 (26.8)		10 (20.4)	12 (28.6)		29 (60.4)	24 (55.8)		7 (12.3)	9 (17.0)		9 (18.4)	10 (22.7)		9 (15.8)	4 (10.5)	
Large selection of produce, *n* (%)																		
Strongly agree, agree, or neutral	177 (67.3)	148 (67.3)	0.99	34 (68.0)	26 (61.9)	0.54	15 (30.0)	16 (37.2)	0.46	47 (81.0)	42 (79.3)	0.81	37 (75.5)	34 (77.3)	0.84	44 (78.6)	30 (79.0)	0.97
Disagree or strongly disagree	86 (32.7)	72 (32.7)		16 (32.0)	16 (38.1)		35 (70.0)	27 (62.8)		11 (19.0)	11 (20.8)		12 (24.5)	10 (22.7)		12 (21.4)	8 (21.0)	
Stressed about food security in the past year, *n* (%)																		
Always, usually, or sometimes	100 (37.6)	98 (44.1)	0.14	21 (42.9)	14 (31.8)	0.28	24 (47.1)	27 (61.4)	0.17	13 (22.4)	20 (37.7)	0.08	21 (41.2)	20 (46.5)	0.61	21 (36.8)	17 (44.7)	0.44
Rarely or never	166 (62.4)	124 (55.9)		28 (57.1)	30 (68.2)		27 (52.9)	17 (38.6)		45 (77.6)	33 (62.3)		30 (58.8)	23 (53.5)		36 (63.2)	21 (55.3)	

^a^ Kruskal–Wallis test statistics produced for continuous metrics, χ2 test statistics produced for categorical metrics.

^b^ USDA standard intake guidelines recommend daily intake of 5+ fruits or vegetables (2015).

USDA, U.S. Department of Agriculture.

#### Neighborhood perceptions of food environment

Unadjusted estimates of our secondary outcomes of interest, both pre and post intervention, are also included in Table 2 for each target neighborhood. A greater proportion of residents from Clairton and Wilmerding reported neutral to positive statements of produce quality and selection post-intervention. Similarly, a greater proportion of Homewood and Mt. Oliver respondents agreed with positive statements of access to produce post-intervention. However, such shifts in the affordability, quality, and selection of produce in the community were not statistically significant. Despite seeing declines in yearly reported food security stress among California-Kirkbride residents, food insecurity did not significantly differ pre–post intervention for any of the neighborhoods.

#### Multivariable analyses

Table 3 displays the crude and adjusted modeling results for our four primary consumption outcomes of interest by neighborhood. In general, the direction and magnitude of pre–post change (expressed in the form of rate ratios with 95% confidence limits) varied by consumption metric and neighborhood.

**Table 3.** Average Change in Produce Consumption Post-intervention (Relative to Pre-intervention) by Neighborhood, Final Adjusted Poisson's Regression Model Rate Ratios (RRs) (Community-Wide Survey, 2015–2017)

Neighborhood	Fruit consumption		Vegetable consumption	
	Days per week^a^	Servings per day^b^	Days per week^c^	Servings per day^d^
	RR (95% CI)	RR (95% CI)	RR (95% CI)	RR (95% CI)
Overall (*n*=490)	0.97 (0.90, 1.03)	^*^0.87 (0.79, 0.97)	1.05 (0.95, 1.16)	0.96 (0.87, 1.07)
California-Kirkbride (*n*=94)	1.04 (0.84, 1.29)	0.85 (0.68, 1.05)	1.06 (0.91, 1.22)	0.97 (0.75, 1.24)
Clairton (*n*=95)	1.08 (0.93, 1.26)	0.87 (0.66, 1.15)	1.01 (0.87, 1.17)	^*^1.20 (1.00, 1.45)
Homewood (*n*=111)	0.94 (0.83, 1.05)	0.93 (0.76, 1.13)	^*^1.13 (1.01, 1.27)	0.86 (0.66, 1.12)
Mt. Oliver (*n*=95)	0.91 (0.82, 1.01)	0.87 (0.69, 1.09)	0.99 (0.84, 1.16)	1.06 (0.88, 1.27)
Wilmerding (*n*=95)	0.87 (0.74, 1.02)	^*^0.83 (0.69, 1.00)	1.06 (0.91, 1.22)	0.80 (0.63, 1.02)

^a^ Model adjusted for marital status and education.

^b^ Model adjusted for gender.

^c^ Model adjusted for gender, seasonality, and education.

^d^ Model adjusted for income.

^*^ Indicates significant estimate at alpha=0.05.

After adjusting for marital status and education level, rates of daily fruit intake (per week) increased by an average of 8% for Clairton residents and 4% for California-Kirkbride residents, post-intervention, but were not statistically significant. Conversely, respondents from Homewood, Mt. Oliver, and Wilmerding experienced declines in this metric. Yet overall, no significant pre–post changes in this outcome measure were observed. With respect to daily number of fruit servings, most neighborhoods observed modest, though insignificant, declines in rates post-intervention. However, after adjusting for gender, Wilmerding residents observed an average 17% decrease in daily number of fruit servings (*p*=0.048).

Of particular significance, pre–post changes in vegetable consumption appeared uniquely meaningful for our target neighborhoods across the board. After adjusting for gender, seasonality, and education level, Homewood residents observed an average 13% increase in their rate of daily vegetable intake post-intervention (*p*=0.04). While not statistically significant, modest increases (of 6%) were also observed for California-Kirkbride and Wilmerding respondents for this metric. Ultimately, covariate adjustment attenuated the magnitude and significance of Mt. Oliver's unadjusted estimate. Finally, after adjusting for income, Clairton residents observed an average 20% increase in their daily rates of vegetable servings post-intervention (*p*=0.049).

### Consumer satisfaction and outcomes survey

Of the 147 consumers recruited to participate in the CSOS, 102 completed the baseline measure and 48 completed the follow-up for cooperation rates of 69% and 47%, respectively, as defined by the AAPOR.^27^ Table 4 displays unadjusted metrics of mobile market satisfaction by *Green Grocer* intervention time. Baseline responses of satisfaction were quite high, with the majority of our sample reporting strong agreement to positive statements of mobile market affordability, quality, and selection. The strength of affirmative responses declined post-intervention, but nonetheless remained positive, with the greatest proportion in somewhat agreement with the three satisfaction metrics. In addition, declines in produce procurement from supercenters and discount stores were observed from pre to post, with subsequent increases in produce purchases from the *Green Grocer* mobile market, farmer's markets, gardens, and other sources.

**Table 4.** Perceptions of Satisfaction with the *Green Grocer* Mobile Market by Intervention Time (Consumer Satisfaction and Outcomes Survey, 2016)

Sample characteristics (*n*=48)	Pre (%)	Post (%)	*p*^a^
*Green Grocer* market is affordable			
Strongly agree	68.8	41.7	0.003
Somewhat agree	27.1	50.0	
Neutral, somewhat, or strongly disagree	4.2	8.3	
Food purchased from *Green Grocer* is of high quality			
Strongly agree	61.7	43.8	0.15
Somewhat agree	31.9	45.8	
Neutral, somewhat, or strongly disagree	6.4	10.4	
Pleased with selection of *Green Grocer* items			
Strongly agree	50.0	33.3	0.26
Somewhat agree	45.8	62.5	
Neutral, somewhat, or strongly disagree	4.2	4.2	
Produce typically purchased most from			
Supermarket, grocery	54.2	52.1	0.11
Supercenter, discount store	31.3	12.5	
*Green grocer*/farmer's market, garden, produce to people	12.5	22.9	
Food pantry, other	2.1	12.5	

^a^ Bowker's test statistics produced for all metrics (H0: symmetric pre–post responses).

## Discussion

This study was designed to evaluate the pilot phase of the important, local *Green Grocer* mobile market program with the goal of addressing food access in low-income communities. Evidence from our CWS highlighted neighborhood-specific improvements. For example, residents of Homewood reported an average 13% increase in vegetable consumption (days/week) post-*Green Grocer* intervention after covariate adjustment (*p*=0.04). Similarly, residents of Clairton observed an average 20% increase in daily vegetable servings (*p*=0.049). Results from our consumer-based survey (CSOS) identified reductions in produce purchasing from supercenters and discount stores alongside purchasing increases from farmer's markets, the *Green Grocer* market, and other sources. Given that the *Green Grocer* mobile market participants reported that they strongly agree or agree that they were satisfied with the market and that we are able to quantify some short-term dietary change, this will provide important data for further expansion to additional geographic areas within and outside of Allegheny County, PA, where this study is located. Many lessons were learned during this time, including understanding the viability of implementing the program in certain sites, timing of visits, design of the mobile truck, zoning issues for parking the mobile truck, and feasibility of surveys. Finally, our study did have some limitations, including lack of a control group and concerns with seasonality, as 6-month follow-up varied by neighborhood, and ranged from August 2016 to January 2017.

When comparing our study with the existing literature on mobile markets,^16,18–20,28^ our results are consistent with findings from other studies that show an increase in perceived food access and some increases in fruit and vegetable consumption. Given the crucial learning experience of this pilot, the GPCFB conducted a full-scale implementation of the *Green Grocer* program in 10 targeted neighborhoods in fall 2017 with a marketing campaign, expansion of sites and educational activities and materials. Our next step in this research is to add a nutrition intervention, a marketing campaign, and educational programming to the *Green Grocer* program in three sites in summer/fall 2018 with the intent of increasing patronage of what the *Green Grocer* has to offer. We will conduct the intervention and pre–post evaluation, and will be able to develop tools for the new project quickly. Our research evaluations, in combination, will provide an opportunity to understand behavioral change in these target communities with the ultimate goal of reducing obesity at the neighborhood level.

## Abbreviations Used

ABS: address-based sampling
ACS: American Community Survey
CSOS: Customer Satisfaction and Outcomes Survey
CWS: community-wide survey
GPCFB: Greater Pittsburgh Community Food Bank
SNAP: Supplemental Nutrition Assistance Program
USDA: U.S. Department of Agriculture
